# Library tools at the nurses' station: exploring information-seeking behaviors and needs of nurses in a war veterans nursing home

**DOI:** 10.5195/jmla.2022.1357

**Published:** 2022-04-01

**Authors:** Gail Kouame, Steph Hendren

**Affiliations:** 1 gailmk.medlib@gmail.com, Associate Professor, Assistant Director for Research & Education Services, Robert B. Greenblatt, M.D. Library, Augusta, GA; 2 stephanie.hendren@duke.edu, Research & Education Librarian, Duke University Medical Center Library & Archives, Durham, NC

**Keywords:** skilled nursing, nursing homes, information seeking, information needs

## Abstract

**Objectives:**

Analyze the information-seeking practices and identify the information and education needs of nurses in a war veterans nursing home. Develop an online toolkit for use at the nurses' stations to meet nurses' health information needs.

**Methods:**

Investigators employed mixed methods to determine the health information needs of the participating nurses at the skilled nursing facility using an online questionnaire and in-person observations. Resulting data was compared to determine how nurses' self-reported data corresponded with investigator observations.

**Results:**

Twenty-seven out of a total of thirty-five nurses responded to the online questionnaire. The study principal investigator also observed a total of twelve nurses working across all shifts. The online questionnaire asked nurses to identify when they need health information for an acute clinical scenario. Nurses self-reported feeling most confident in assessing falls and pain, followed by medication adherence and skin integrity. Issues most frequently encountered during observation of nurses were falls, interventions surrounding cognitive ability or dementia, and use of antibiotics. Nurses reported and were observed to consult colleagues most frequently, followed by drug handbooks and relying on nursing experience.

**Conclusion:**

Nurses in skilled nursing facilities will benefit from ready online access to current drug handbooks as well as information resources surrounding commonly encountered clinical issues and stated needs. An outcome of this project is an online toolkit site using a LibGuide created specifically for this purpose. Researchers purchased laptop computers that were installed at each of the nurses' stations to provide ready access to the toolkit site.

## INTRODUCTION

Nurses in skilled nursing facilities care for older patients who have multiple diagnoses, take numerous medications, are physically frail, and are often cognitively impaired. Often, there is no physician on-site with whom to consult, provide treatment recommendations, or give orders for patient care. When acute patient care issues arise, nurses assess the situation and determine whether treatment or intervention may be required. Cohen-Mansfield, Lipson, and Horton note that “the nursing staff spends much more time [than physicians] with residents, attends to their functional and self-maintenance needs, and alerts physicians to medical needs and concerns” [1]. Most needed treatments must be prescribed by a physician or other health professional with prescriptive abilities, such as an advanced registered nurse practitioner or physician assistant. In addition, as noted by Kogan et al., “Medicare mandates oversight by a physician for developing and implementing multidisciplinary care plans” [2]. In order to perform an adequate assessment and to convey accurate information to the physician or other provider with oversight, nurses turn to a variety of information sources. The principal investigator on this study previously worked as a social worker in skilled nursing facilities and observed nurses' interactions with physicians and other prescribers when confronted with acute care situations. She formed research questions about how nurses in skilled nursing facilities seek information and how the nurses prepare to relay information to other care providers for patient care orders.

Multiple studies have been published about nurses' experiences in the long-term care setting, including physician-nurse communication dynamics, leadership and management issues, and process and quality improvement initiatives. To date, no literature has been found that describes how nurses in skilled nursing facilities seek information to inform interactions with physicians and other providers to implement care plans for patients with acute health care needs. One study found that nurse preparedness for communication with physicians was a contributing factor to the quality of physician encounters. A nurse who participated in the study is quoted as saying, “I think if you're prepared and have all the information needed when you talk to a doctor, it makes it go that much smoother” [3].

The purpose of the study performed at the Georgia War Veterans Nursing Home (GWV) was to analyze the information-seeking practices of nurses in a skilled nursing facility, as well as to determine the nurses' health information needs, both perceived and observed, when faced with an acute patient care situation. In the context of this study, the acute patient care situation serves as the information need perceived by an information seeker, the nurse, as outlined in T. D. Wilson's model of information behavior. According to Wilson, information seekers, when faced with an information need, will turn to either formal or informal information sources or services to find relevant information. Wilson's model allows for the possibility that

part of the information-seeking behavior may involve other people through information exchange and that information perceived as useful may be passed to other people, as well as being used (or instead of being used) by the person himself or herself [4].

Study investigators recognized this possibility and incorporated questions in the online questionnaire, as well as on the observation checklist, that allowed for reliance on professional colleagues as information sources. The results of the study helped to inform the contents of an online toolkit to be used by the nurses at GWV that was a planned outcome of the project.

## METHODS

### Study setting

The setting for this study was the GWV in Augusta, GA. Operated through an interagency agreement with Augusta University and funded by the state of Georgia, the GWV is a 192-bed skilled nursing facility adjacent to the Augusta University health sciences campus. Primarily, the facility provides medical and nursing services to Georgia's aged and infirm veterans. The average age at the GWV is eighty-four. Unlike many skilled nursing facilities, the medical director, an MD, is in-house at the GWV. There are also several physician assistants and medical residents who participate in clinical rotations at the facility. A pharmacist and pharmacy students are also frequently on-site. There are thirty-five licensed nurses, including nurse administrators, who work at the facility across three shifts. The nurse managers and nurse administrators (director of nursing, assistant director of nursing, nurse educator, and minimum data set coordinators) work Monday through Friday from eight o'clock in the morning until five o'clock in the afternoon. On the day shift, there are two licensed practical nurses on each floor plus a registered nurse house supervisor, along with a nurse manager. On the evening and night shifts, there is one licensed practical nurse and one house supervisor per floor. The nurse managers and nurse administrators are available for part of the evening shift. On the night shift, there is one licensed practical nurse and house supervisor per floor. Certified nursing assistants work under the direction and oversight of the licensed nurses.

There are desktop computers at each of the nurses' stations, but they are located in busy work spaces and are primarily for use to monitor patients with bed or chair alarms who are at a high risk to fall. There are work rooms located behind each nursing station that provide a quieter area where nursing staff can look up information or focus on making entries in patient charts. Having information resources available through an online toolkit on computers in the work rooms would allow for a more efficient way of accessing needed information. Prior to this study, nurses relied on print drug handbooks and other print materials that were sometimes out of date.

### Study preparation

This study was reviewed and approved for exempt status by Augusta University's Institutional Review Board. Researchers met several times with the medical director, the director of nursing, the assistant director of nursing, and the nurse educator at the GWV to introduce and describe the study. One goal of these meetings was to get expert input about nurses' commonly encountered clinical situations in order to inform the development of the online questionnaire as well as the content of the online toolkit site.

### Study population

The target population of this study was the licensed nurses who provide patient care at the GWV in Augusta, GA. Eligible participants in the study were licensed registered nurses or licensed practical nurses employed by the GWV. The nurse educator assisted with advertising the study by sending emails to all the nurses and by posting flyers in staff areas at the nursing home. She also set up an introductory session for the nurses where researchers explained the study to the nurses, and the nurses were able to ask questions about the study. The nurse educator videotaped the session and made it available to nurses who were unable to attend, such as those who worked night shift.

### Quantitative data collection

**Figure 1:**
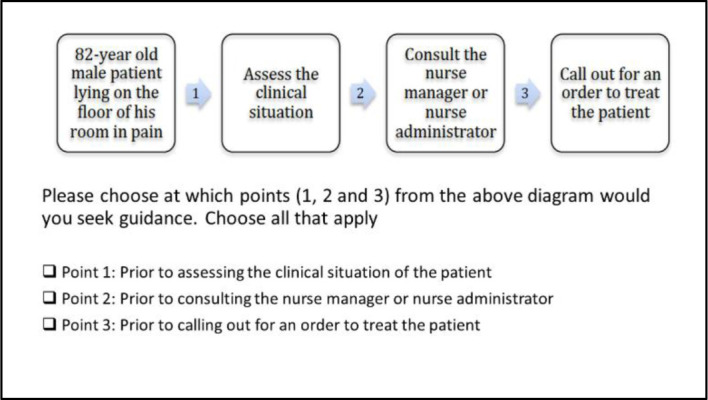
Information-seeking flowchart

Researchers employed a biostatistician from the Augusta University Department of Population Health to assist with developing the questionnaire and to interpret the resulting data. Participating nurses received a link to an online consent form, followed by the online questionnaire (Appendix A). The online consent and questionnaire were administered using Qualtrics survey software. No individual identifying information was collected. The questionnaire opened with a series of questions to collect demographic data as well as the length of time respondents have worked as a nurse. It then assessed at what points in a patient care scenario the nurses consult information (Figure 1).

A question followed about what types of information sources nurses consult in their work. The nurses could choose all the appropriate information sources from the list provided. The list was created based on input from the nurse leadership team at GWV as well as resources available through the Augusta University Libraries. The questionnaire concluded by asking nurses to rate their confidence level in assessing commonly encountered patient issues in the long-term care setting such as falls, skin integrity, mental health issues, and urinary tract infections.

### Participant observation data collection

In addition to the online questionnaire, the principal investigator for the project observed nurses on each of the four floors of the GWV across all three shifts (day shift, swing shift, and night shift), for a total of twelve observations. This mixed methods approach was chosen because, as Reddy and Spencer state,

since people often cannot tell a researcher what they actually do in practice (rather than what they are supposed to do), it has been found more useful to both interview and observe study participants [5].

The intent of the observations was to analyze when and how the nurses seek information to support clinical decisions when they encountered an acute clinical care situation. The researcher employed a form with a checklist of common health issues and also asked questions of the nurses during clinical encounters as needed (Appendix B).

The observation checklist started with general demographic questions about the participants as well as which shift was being observed. It then asked observers to indicate at which points during a patient clinical encounter the nurse sought guidance from information sources. Following that was a checklist of information resources the nurse could possibly consult, including an “other” option to account for any sources not listed. Next was a list of possible common issues that could be encountered, as well as an option for “other” to allow for situations not covered by the list provided.

## RESULTS

### Quantitative data analysis

Twenty-seven of the thirty-five nurses employed by the GWV responded to the online survey, a response rate of 77%. Respondent characteristics are presented in Table 1. Nurses at the GWV have many years of nursing experience. The majority of the nurses were aged fifty-five to sixty-four years (n=15, 57%) and had been working as a nurse for twenty-five to twenty-nine years (n=8, 29%), followed closely by nurses who had been working for thirty to thirty-four years (n=6, 22%). A follow-up question asked how long the nurses had worked in skilled nursing care. The majority of the twenty-five respondents to this question (n=8, 32%) had worked in skilled nursing for ten to fourteen years. Six nurses, or 24% of those who responded, had been working in skilled nursing for twenty to twenty-four years.

**Table 1:** Respondent characteristics

Gender		Age (years)		Length of time as a nurse (years)		Length of time worked in skilled nursing care (years)		Distribution of work shift	
**Total**	**27**	**Total**	**27**	**n**	**27**	**n**	**24**	**n**	**27**
Male	0	18 to 24	0	0 to 2	3	0 to 2	2	Days	20
Female	26	25 to 34	1	3 to 9	0	3 to 9	2	Evenings	6
Prefer not to answer	1	35 to 44	4	10 to 14	4	10 to 14	8	Nights	4
		45 to 54	7	15 to 19	1	15 to 19	2	Other (weekends, on-call)	4
		55 to 64	15	20 to 24	3	20 to 24	5		
		65 and older	0	25 to 29	8	25 to 29	2		
				30 to 34	6	30 to 34	1		
				35 to 39	1	35 to 39	1		
				40 to 44	1	40 to 44	1		
				45 to 49	0	45 to 49	0		

When presented with an example clinical scenario, the majority of respondents (n=14, 52%) indicated they would need to access information after encountering an acute clinical situation and prior to assessing the clinical situation. They also reported seeking information prior to assessing patient clinical status (n= 14, 52%), consulting the nurse manager (n=8, 28%), and calling for treatment orders (n=10, 36%).

In terms of the types of information sources consulted in their job, the largest number of nurses self-reported that they turn to clinical staff such as physicians, pharmacists, or therapists. A very close second was nursing colleagues as sources of information. The third most popular source of information was drug handbooks.

Assessment of falls and pain were each chosen five times as areas in which nurses felt most confident. Assessment of cognitive ability and cardiovascular health were each chosen four times as issues that the nurses felt confident assessing. Medication adherence (n=2, 7%) and mental health (n=1, 3%) followed as areas in which nurses felt confident.

### Participant observation data analysis

The principal investigator observed nurses dealing with a variety of clinical situations across three shifts. The nurses at the GWV are accustomed to being observed and talking with other people about what they do because they are frequently accompanied by nursing and other health professions students performing geriatric clinical rotations. In this study, clinical issues most commonly encountered and requiring ongoing nursing assessment during observations were patient falls, issues surrounding impaired cognitive abilities (or changes in cognitive status), and antibiotic treatments. These three situations were observed six times each during the twelve observation periods. The next most commonly observed issues were patients with urinary tract infections and patients requiring skin treatments. There were four incidents of each during the twelve observation periods. Three patient encounters involved tube feedings, and two patients had eye infections that required treatment and observation. The observational data recorded nurses seeking information at different frequencies for assessing patient clinical status (n=2, 16%), consulting the nurse manager (n=10, 83%), and calling for treatment orders (n=8, 66%).

The nurses turned most frequently to nursing colleagues as information sources when questions arose about a plan of care for particular patient situations. This was observed seven times during the twelve observations. The next most common source of information was the nurses' own nursing experience. The investigator observed this five times. On one occasion, a nurse consulted with a physician, and in another situation a nurse referred to a protocol or guideline during the observation period.

## DISCUSSION

There was a correlation between the health issues that the nurses at the GWV expressed confidence in assessing in the online questionnaire and those that were observed by the principal investigator of this study. Notably, falls and cognitive ability were among the top three topics in both the nurses' self-reporting and in what the researcher observed. Also, due to the extensive years of nursing experience represented by the nurses at the GWV, it is logical that they rely heavily on that experience when they encounter common clinical scenarios. Because elderly patients are often on multiple medications, it is reasonable that drug handbooks would be among the top information sources that nurses report using.

Based on the principal investigator's past professional experiences and observations in long-term care facilities, it was somewhat unexpected that nurses report needing to access information immediately after encountering an acute clinical situation and before assessing the situation. Less surprising was the finding that nurses more commonly need to access information after they've consulted with a nurse manager and before calling upon a physician or other health care provider for a treatment order or other intervention.

The nurses' frequent use of professional colleagues as information sources is consistent with the findings of several studies of nurses and health professionals in other clinical settings [6–12]. In each of these studies, lack of time was the primary motivator for information seekers to consult other people, as opposed to other information sources, even when those sources were readily available. Additionally, one study performed in a skilled nursing facility (SNF) regarding heart failure management found that “SNF staff and physicians rely on verbal communication without tools to assist or track communication” [13]. As noted by Katriina Bystrom in her theory of information activities in work tasks, “as soon as information acquisition requires an effort people as sources become more popular than documentary sources” [14].

### The online toolkit

**Figure 2:**
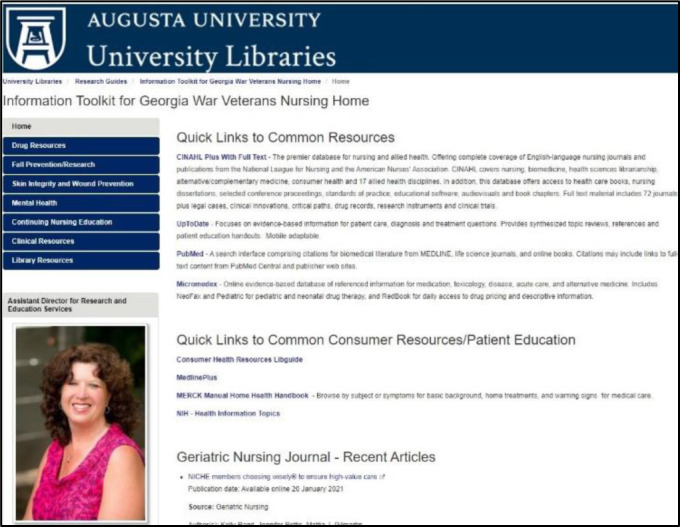
Information toolkit home page

Based on the quantitative and observational findings of this study, as well as input from the leadership at the GWV, the project team developed a web-based toolkit with links to information resources relevant to caring for the geriatric population (Figure 2) [15]. The home page of the toolkit provides links to general information about the Augusta University Health Sciences library as well as to general literature and drug databases such as CINAHL, PubMed, and Micromedex. There is also an RSS feed to provide current citations to the journal *Geriatric Nursing*, to which the library subscribes.

Beyond the home page, the tabs are organized based on the common health issues that nurses frequently treat or need information about, such as drugs and vaccinations, fall prevention and research, skin integrity and wound prevention, and mental health. The nurse administrators asked specifically to include information about vaccinations, as this is an area about which the staff nurses need to keep current. In the topic areas of fall prevention and research and mental health, librarians from the Greenblatt Library at Augusta University created preconfigured searches in relevant databases, so the nurses can access the current journal literature on the topic with just one click.

Additionally, nurses can access free continuing education resources through the CINAHL database and through the Centers for Disease Control and Prevention. Along with informing the nurses for their day-to-day duties, the toolkit also aims to provide resources for patient and family education by providing links to consumer health information resources for each topic through MedlinePlus. As of this writing, the toolkit has been viewed 271 times since it was launched in July 2017. As mentioned previously, laptops were installed at each nurses' station, which allows for the nurses to access the information resources on the units so the nurses do not have to leave the patient care area.

Information regarding the results of the project and the final LibGuide was communicated in a scheduled meeting where the librarians demonstrated the resources and how they could be used. The GWV nurse educator recorded the session and made it a requirement to watch the session so all nurses were aware of the new resource. Because of the GWV's affiliation with Augusta University, employees of the GWV can access the university libraries' resources for the health sciences campus. Prior to this project, many of the nursing staff were not aware they had this benefit. They were pleased to learn that they can access so many resources readily. The director of nursing was particularly proud of the ability to link out to the most current drug handbooks, as having access to this information is something state nursing home inspectors require.

## LIMITATIONS

The GWV is unique in that the medical director is in-house and because of its affiliation with a nearby health sciences university. An additional aspect of the university affiliation is that the nurses have access to the university health sciences library's information resources. Due to this distinctive environment, this study is likely not generalizable to other nursing homes or long-term care facilities. In more traditional long-term care settings, the medical director and other primary health care providers are offsite and nurses are compelled to interact by telephone and other means more regularly. Additionally, the length of service of the nurses at the GWV is high (twenty of twenty-four respondents have worked in skilled nursing for at least ten years), so the findings of this study may be skewed to reflect more reliance on nursing expertise than would be found in other facilities. Lastly, the residents of the GWV are long-term residents and are not expected to be discharged back home or to lesser care settings in the community. In other nursing home settings, this is not usually the case in that residents may be admitted for short-term rehabilitation services or transferred to another facility for a variety of reasons. The nurses at the GWV have been caring for their patients for an extended time and are quite familiar with the patients' patterns and needs. These dynamics also likely contributed to the nurses relying on one another and on their own expertise.

## CONCLUSION

This study took a unique look at how nurses in long-term care prepare for communication with physicians when they encounter an acute clinical care situation with patients. Prior to this study, the authors found no comparable literature exploring the information-seeking needs and behavior of nurses in this particular setting. An area for possible future research would be to do a similar study in a skilled nursing facility setting that is not affiliated with an academic institution. In nonacademic settings where the medical director and other physicians or prescribers are not on-site, there are additional challenges for nurses in preparing to relay patient information during an acute clinical issue.

The findings reflected in the data from this study confirm that long-term care nurses rely on their nursing experience and on their professional colleagues as primary sources of information. These findings are consistent with T. D. Wilson's model of information behavior, which acknowledges that information seekers, when faced with an information need, may use informal information and services as well as formal ones [4]. One example of the nurses using formal information resources was their regular use of drug handbooks. Making electronic drug handbooks and other information resources more readily accessible through the online toolkit that was developed for this project is a benefit to the nurses and will help the GWV stay current with state standards. An added benefit of the toolkit is ready access to free continuing education modules and credit provided by one of the database providers and by the Centers for Disease Control and Prevention. This project also raised awareness among the nurses at the GWV about their eligibility to access resources through the Robert B. Greenblatt, M.D. Library at Augusta University because of the nursing home's affiliation with the university.

## Data Availability

Data associated with this article are available in the Open Science Framework at https://augusta.openrepository.com/handle/10675.2/624125.

## SUPPLEMENTAL FILES

**Appendix A.** Online consent and questionnaire

**Appendix B.** Observation survey
