# Supplementary material for: Library tools at the nurses' station: exploring information-seeking behaviors and needs of nurses in a war veterans nursing home
Source: J Med Libr Assoc. 2022 Apr 1;110(2):159–65. doi: 10.5195/jmla.2022.1357 (PMC9014917; doi:10.5195/jmla.2022.1357)
Supplement: Supplementary file 1 — Appendix A. Online consent and questionnaire [file jmla-110-2-159-s01.pdf]

Appendix A

Online Consent and Questionnaire

Consent

You are being invited to participate in a research project to study the health information seeking behaviors and needs of nurses in skilled nursing facilities because you are an employee at the Georgia War Veterans Nursing Home. This research project is being funded by the National Library of Medicine. The survey asks questions about how and when you access health information to support clinical decisions. It should take you about 10 minutes to complete.

The results of this project will be used for developing a web-based health information toolkit for your use at the Georgia War Veterans Nursing Home and for informing others working in long-term care how to support their nurses with their health information needs. Through your participation I hope to understand when you need to consult information resources, and what information resources you consult to help you make decisions. I hope that the results of the survey will be useful for other information professionals as well as others working in skilled nursing facilities. I hope to share my results by publishing them in the professional literature as well as giving presentations about them at professional conferences attended by other librarians.

There are no known risks to you if you decide to participate in this survey. There is no direct benefit to you for participating in this study. The alternative would be not participating in the study. I will not share any information that identifies you with anyone outside my research group which consists of me and Dr. Frances Yang.

I will do my best to keep your information confidential. All data is stored in a password protected electronic format. To help protect your confidentiality, the surveys will not contain information that will personally identify you. The results of this study will be used for scholarly purposes only and may be shared with Augusta University representatives.

I hope you will take the time to complete this questionnaire; however, if you agree to complete the survey you are not required to answer all the questions or complete it. Your participation is voluntary and there is no penalty if you do not participate. If you have any questions or concerns about completing the questionnaire, about being in this study, or to receive a summary of my findings you may contact me at (706) 721-9904 or gkouame@augusta.edu.

If you have any questions or concerns about the "rights of research subjects," you may contact the Augusta University IRB Office at (706) 721-1483.

Sincerely,

Gail Kouame, MLIS

Greenblatt Library

1120 15th Ave. – AB 241

Augusta, GA 30912

Clicking on the "agree" button below indicates that:

- **you have read the above information**
- **you voluntarily agree to participate**
- **you are at least 18 years of age**

If you do not wish to participate in the research study, please decline participation by clicking on the "disagree" button.

- Agree
- Disagree

<u>Questionnaire</u>

Q1: Are you?

- Male
- Female
- Prefer not to answer

Q2: What is your age? Please choose a category from below:

- 18 to 24 years
- 25 to 34 years
- 35 to 44 years
- 45 to 54 years
- 55 to 64 years
- 65 years and older

Q3: How long have you been a nurse? Please choose a category from below:

- 0 to 2 years
- 3 to 9 years
- 10 to 14 years
- 15 to 19 years
- 20 to 24 years
- 25 to 29 years
- 30 to 34 years
- 35 to 39 years
- 40 to 44 years
- 45 to 49 years

Q4: How long have you worked in skilled nursing care? Please choose a category form below:

- 0 to 2 years
- 3 to 9 years
- 10 to 14 years
- 15 to 19 years
- 20 to 24 years
- 25 to 29 years
- 30 to 34 years
- 35 to 39 years
- 40 to 44 years
- 45 to 49 years

Q5: Which shift do you work?

- Days
- Evenings
- Nights
- Other (such as, weekends, on-call): please type in ______________

Q6: You have an 82-year old patient who is found lying on the floor of his room in pain. Below is a diagram with numbered points for acquiring information. At which point do you need to access information?

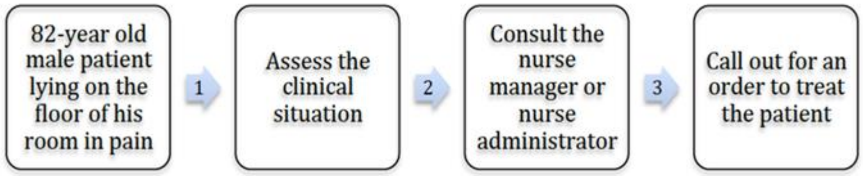

Please choose at which points (1, 2, and 3) from the above diagram would you seek guidance. Choose all that apply.

- Point 1: Prior to assessing the clinical situation of the patient
- Point 2: Prior to consulting the nurse manager or nurse administrator
- Point 3: Prior to calling out for an order to treat the patient

Q7: What information resources do you currently consult in your day to day work? Please select all that apply.

- Nursing colleagues
- Other clinical staff (such as physicians, pharmacists, or therapists)
- Drug handbook
- Mosby’s
- Search the Internet (such as, Google)
- Consult a known health-related website (such as, Medline Plus)
- Library resources at Augusta University
- Other: Please type in a resource that you use ________________________

Q8: In general, what is your confidence level for assessing the following areas regarding patient care? Rank order the following 10 topics of patient care with an additional "Other" topic for you to write in. Select and hold the topic you feel most confident about and move it to the top of the list, as number 1, let go of the topic after you placed it. Then continue with the next topic that you feel relatively less confident about, and move it to number 2, until you reach your least confident topic at the bottom of the list.

- Falls
- Mental health
- Oral care
- Skin integrity
- Urinary tract infection
- Pain
- Medication Adherence
- Cognitive ability
- Cardiovascular health
- Vaccinations
- Other: Please type in a topic and move it to the position for your confidence level (top is most confident, bottom is least confident) ______________________
