# Supplementary material for: Library tools at the nurses' station: exploring information-seeking behaviors and needs of nurses in a war veterans nursing home
Source: J Med Libr Assoc. 2022 Apr 1;110(2):159–65. doi: 10.5195/jmla.2022.1357 (PMC9014917; doi:10.5195/jmla.2022.1357)
Supplement: Supplementary file 2 — Appendix B. Observation survey [file jmla-110-2-159-s02.pdf]

Appendix B
Observation Survey

Assessing the Health Information Seeking Behaviors and Needs of Nurses in Skilled Nursing Facilities

SURVEY/CHECKLIST – *To be used while observing nurses during their shifts at the Georgia War Veterans Nursing Home*

Q1: Is the nurse:

- Male
- Female
- Prefer not to answer

Q2: Ask the nurse: What is your age? Check the appropriate category from below:

- 18 to 24 years
- 25 to 34 years
- 35 to 44 years
- 45 to 54 years
- 55 to 64 years
- 65 years and older

Q3: Ask: How long have you been a nurse? Check the appropriate category from below:

- 0 to 2 years
- 3 to 9 years
- 10 to 14 years
- 15 to 19 years
- 20 to 24 years
- 25 to 29 years
- 30 to 34 years
- 35 to 39 years
- 40 to 44 years
- 45 to 49 years

Q4: Ask: How long have you worked in skilled nursing care? Check the appropriate category from below:

- 0 to 2 years
- 3 to 9 years
- 10 to 14 years
- 15 to 19 years
- 20 to 24 years
- 25 to 29 years

- 30 to 34 years
- 35 to 39 years
- 40 to 44 years
- 45 to 49 years

Q5: Which shift is being observed?

- Days
- Evenings
- Nights

Q6: Please choose at which points (1, 2, and 3) during a patient clinical encounter the nurse seeks guidance from information sources. Choose all that apply.

- Point 1: Prior to assessing the clinical situation of the patient
- Point 2: After assessing the clinical situation and prior to consulting the nurse manager or nurse administrator
- Point 3: After consulting the nurse manager or administration and prior to calling out for an order to treat the patient.

Q7: What information resources did the nurse consult in the clinical encounter?

- Colleagues
- Drug handbook
- Mosby's
- Search the Internet (such as, Google)
- Consult a known health-related website (such as, Medline Plus)
- Library resources at Augusta University
- Other: Please type in a resource that you use
  ____

Q8: Which topics were encountered during the nurses' clinical encounters with patients during their shift?

- Falls
- Mental Health
- Oral Care
- Skin Integrity
- Urinary Tract Infection
- Pain
- Medication Adherence
- Cognitive Ability
- Cardiovascular Health
- Vaccinations
- Other:
